# Intradermal delivery of SARS-CoV-2 RBD3-Fc mRNA vaccines via a needle-free injection system induces robust immune responses in rats

**DOI:** 10.3389/fimmu.2025.1530736

**Published:** 2025-02-17

**Authors:** Cenrong Wang, Xin Tang, Chenghan Jiang, Yu Zhang, Bo Han, Yi Sun, Jianfeng Guo, Hanyu Peng, Zihan Wang, Yipeng Wang, Jialu Zhang, Yong Zhang, Chunlai Jiang

**Affiliations:** ^1^ National Engineering Laboratory for AIDS Vaccine, School of Life Sciences, Jilin University, Changchun, China; ^2^ R&D Department, Changchun BCHT Biotechnology Co., Changchun, China; ^3^ College of Agriculture, Yanbian University, Yanbian, China; ^4^ R&D Department, Jiangsu Leju Medical Technology Co., Jiangsu, China; ^5^ Key Laboratory for Molecular Enzymology and Engineering, the Ministry of Education, School of Life Sciences, Jilin University, Changchun, China

**Keywords:** needle-free injection system, intradermal injection, inoculation routes, mRNA vaccines, SARS-CoV-2

## Abstract

**Introduction:**

Needle-free injection system (NFIS) is easy to operate and can decrease needle phobia. Besides, NFIS can increase the interaction of antigens in a more dispersed manner with immune cell at local injection site, which may improve the immune responses of mRNA vaccines. Although SARS-CoV-2 mRNA vaccines have great success, universal vaccines are urgently needed. Delivering universal mRNA vaccines by NFIS is preferred to combat COVID-19.

**Methods:**

RBD3-Fc mRNA expressing BA.4, Delta, and prototype RBD, and human IgG Fc with YTE mutation was designed and synthesized. The safety and immune responses of universal RBD3-Fc naked mRNA and mRNA-LNP vaccines delivered intradermally using NFIS (named GV-01) and intramuscularly via needles were evaluated and compared in rats.

**Results:**

The prime-boost regimen administered by two routes resulted in potent immune responses and intradermal delivery displays comparable or better performance in terms of binding antibodies, neutralizing antibodies and T cell responses. Naked mRNA vaccines were functional, but less effective than mRNA-LNP vaccines.

**Discussion:**

The above results suggest that RBD3-Fc vaccines are safe and immunogenic and NFIS can be used as an alternative to needles/syringes for the inoculation of mRNA-LNP vaccines to elicit robust systematic immune responses.

## Introduction

A needle-free injection system (NFIS) was developed in the 1930s to combat epidemics (1). NFIS can deliver vaccines into the desired tissues via a high-velocity jet stream through a narrow orifice driven by high pressure (e.g., intramuscular (IM), intradermal (ID), and subcutaneous (SC) vaccination) (1–3). Compared to traditional needle injection, which usually leads to a bulk of liquid, the NFIS often delivers the medicine in a more dispersed manner, which increases its exposure to cells [e.g., macrophages, Langerhans cells (LCs), and dendritic cells (DCs)] at the injection site. As a result, antigen presentation can be promoted when delivered via the NFIS (4, 5), which may be a better choice for mRNA vaccine delivery. Compared to traditional needles, the NFIS is easy to operate and can decrease the use of needles and the need for sharps disposal, which incurs significant medical costs (6, 7). Moreover, the use of the NFIS significantly avoids needle phobia, especially in children, which affects at least 10% of the general population (1, 8, 9). In a survey by Tropis, 94.7% of caregivers indicated that they would be more likely to take their children for vaccination in future campaigns using NFIS instead of traditional needles and syringes (10). For these reasons, the NFIS offers remarkable advantages for managing new outbreaks by vaccinations: improved safety, better compliance, decreased or eliminated injection site pain, easier and speedier vaccine delivery, and reduced cost (11).

Administration routes influence vaccine efficacy, particularly the magnitude, quality, and persistence of the immune response (12). The viable epidermis and dermis contain many antigen presenting cells (APCs) that can capture antigens and subsequently migrate to the draining lymph nodes to trigger systemic immune response. Besides, APCs and epidermal keratinocytes can also produce cytokines and chemokines like TNF-α and IL-1β to promote antigen presentation and migration (13). It is reported that in some cases, nucleic acid vaccines via the NFIS by different routes exhibit comparable or higher immunogenicity and dose-sparing effects than traditional injections (5, 14–17). However, whether the efficacy and immunogenicity of mRNA vaccines intradermally delivered by NFIS in the real world are better than those delivered by needle injections remain unclear, which more trials are required to determine the extensive application potential of the NFIS in mRNA vaccines. Currently, the PharmaJet Stratis injector can deliver vaccines via the IM and SC routes and the Tropis injector for the ID route has also been approved by regulatory agencies as a spring-powered device (1, 3, 17). However, both are rarely applied for mRNA vaccine delivery. Thus, further investigation is needed to illustrate the suitability of NFIS for mRNA vaccine delivery.

With the continuous evolution of SARS-CoV-2, an effective universal vaccine against variants of concern (VOC), especially Omicron sub-lineages (BA.1, BA.2, and BA.4/5) (18–20), whose outbreak has led to more than 776 million reported cases and 7.07 million deaths (https://covid19.who.int/) (21), must be urgently developed. mRNA vaccines, acknowledged to be a safe and efficient vaccine platform against epidemics (e.g., BNT162b2 and mRNA-1273 (22, 23), have been shown to easily adapt to viral mutations owing to their quick production and convenient construction directly from genetic sequence information (24, 25). To design universal vaccines, receptor-binding domain (RBD) of S protein is usually chosen as an immunodominant epitope for inducing high and potent neutralizing antibodies (nAbs) (26–29). S protein (~150 kD), existing as a homotrimer in the virus envelope, consists of S1 and S2 and can be cleaved by host furin-like proteases. RBD, part of S1 subunit, binds to the human angiotensin-converting enzyme 2 (hACE2) receptor, and the S2 subunit is required for viral entry and membrane fusion (27, 30–33). Of note, RBD may avoid the potential risk of antibody-dependent enhancement (ADE) (34, 35).

In this study, a SARS-CoV-2 mRNA that expressed three RBD domains and a human IgG Fc fragment with YTE mutation was synthesized and encapsulated in lipid nanoparticles (LNP). A gas-propelled NFIS of GV-01 was then calibrated and its ID delivery capacity was confirmed. Different doses of the mRNA-LNP vaccine were administered to rats via the IM route with needles or ID route using GV-01, and the elicited immune responses were evaluated and compared. The potential of naked mRNA vaccine without LNP that can be directly injected for translating antigens was also determined. ID administration of the mRNA-LNP vaccine using GV-01 displayed comparable or better performance than IM administration in terms of safety, binding antibody, and neutralizing antibodies. Moreover, vaccination with mRNA-LNP via the ID route induced a more robust CD8+ T-cell response than that via the IM route. Naked mRNA vaccines exhibited their functions when administered via the ID or IM route, but were less effective than the mRNA-LNP vaccine. This study confirmed that both RBD3-Fc naked mRNA and mRNA-LNP vaccines are safe and immunogenic, and that the NFIS of GV-01 could be used as an alternative for inoculating either naked mRNA or mRNA-LNP vaccines.

## Materials and methods

### Compliance statement

The animal studies were performed in accordance with the Guide for the Care and Use of Laboratory Animals (National Research Council). All animal procedures were reviewed and approved by the Animal Welfare and Research Ethics Committee of Changchun BCHT Biotechnology Co. (No. BCHT- AEEI-2023-157 and BCHT- AEEI-2023-451). All animals were euthanized using euthanasia systems by inhaling CO_2_ after all experiments.

### Experimental animals

Specific pathogen-free female SD rats (age, 6-7 weeks; weight, 160–200 g) were purchased from Liaoning Changsheng Biotechnology Co., Ltd. (Liaoning, China) and maintained under standard approved conditions. The rats acclimated to the lab for 3-5 days were vaccinated until weights of 220-240 g (aged 9-10 weeks) were achieved, which was available to inject by GV-01 with applicable skin thickness.

### Vaccine procedure

To ensure that GV-01 precisely delivered the vaccine intradermally, we mixed the vaccines with the indicator, rhodamine. Following injection using the optimized PSI parameters, the rats (n = 2) were sacrificed, and the corresponding tissues were collected and photographed for the initial proof-of-concept.

Rats were randomly divided into 10 groups (**Table 1**). Four groups were inoculated with a series dose of mRNA-LNP (30, 50 μg) and mRNA (30, 50 μg) per rat, respectively, via the IM route. Other groups were vaccinated by GV-01 with same doses. Unvaccinated control animals were administered an equivalent volume (100 μL) of LNP buffer. Vaccination was performed on days 0 and 15. Sera were collected 2, 4 (n = 6) and 6 (n = 3) weeks after each vaccination in all groups. The spleens (n = 3) for ELISpot detection and Single-cell analysis were collected four weeks after the boost.

**Table 1:** Grouping scheme.

Group	Vaccine	Dose	Injection site	Injection volume	Immunization route
1	LNP buffer	None	Leg	100 µL	IM
2	mRNA-LNP	30 µg	Leg	100 µL	IM
3	mRNA-LNP	50 µg	Leg	100 µL	IM
4	mRNA	30 µg	Leg	100 µL	IM
5	mRNA	50 µg	Leg	100 µL	IM
6	LNP buffer	None	Back	100 µL	ID by GV-01
7	mRNA-LNP	30 µg	Back	100 µL	ID by GV-01
8	mRNA-LNP	50 µg	Back	100 µL	ID by GV-01
9	mRNA	30 µg	Back	100 µL	ID by GV-01
10	mRNA	50 µg	Back	100 µL	ID by GV-01

### mRNA synthesis and purification

**Figure 1:**
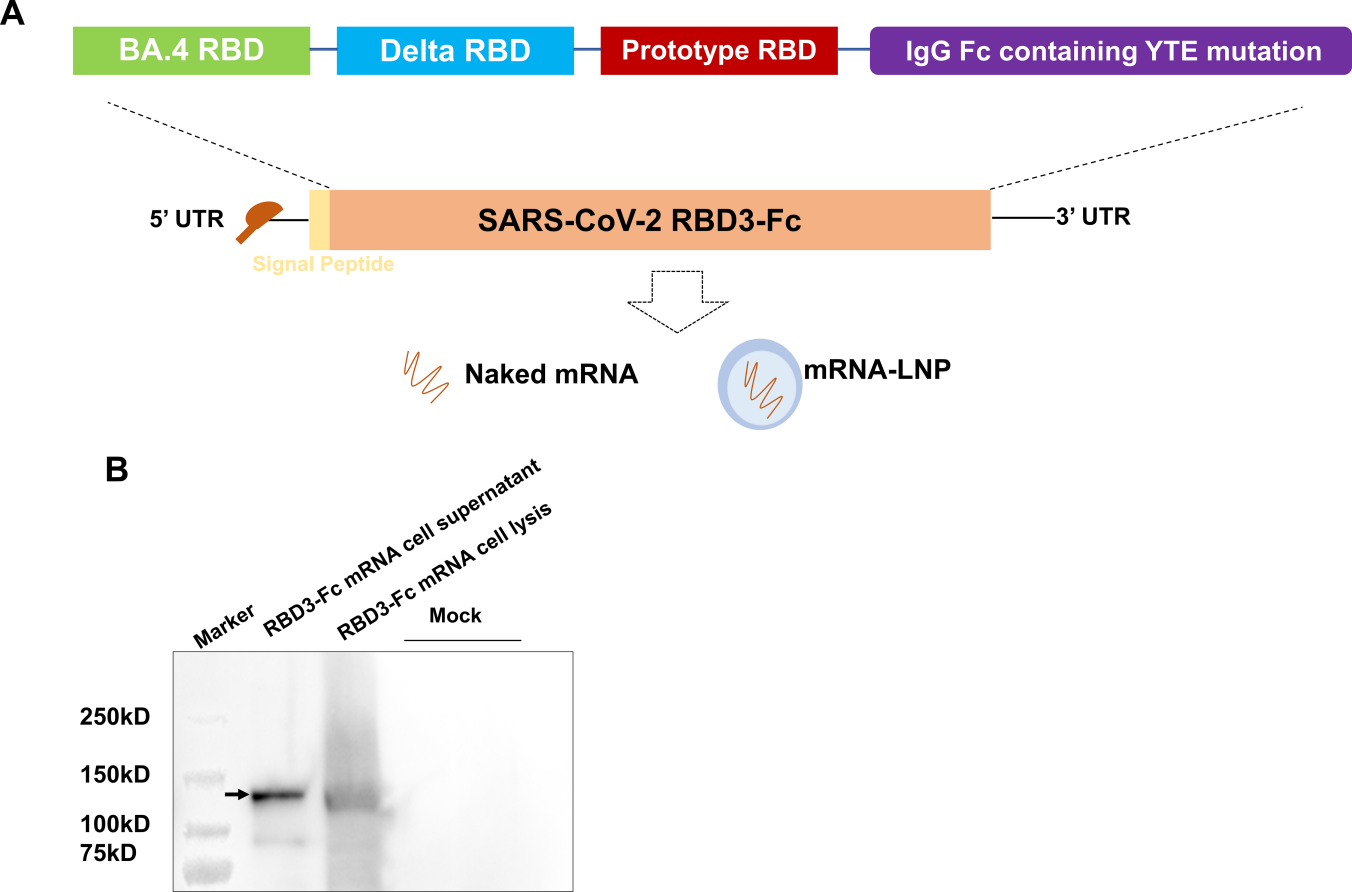
Design and characterization of the RBD3-Fc mRNA vaccine candidate. **(A)** Schematic of RBD3-Fc mRNA. **(B)** Protein expression of RBD3-Fc mRNA in HEK293T cells detected via western blot. Data are representative of one independent experiment.

RBD3-Fc mRNA expressing BA.4 RBD, Delta (B.1.617.2) RBD, prototype RBD (D614G strain), and human IgG Fc containing YTE mutation was designed as illustrated in **Figure 1A**, and comprised 5’ Cap1 structure, 5’UTR, coding sequence, 3’UTR structure, and poly A tail. Briefly, 50 µg/mL linear plasmid (Genscript Biotech Co., China) was used as the template for an IVT reaction at 37°C for 6 h with ribonucleotides and 2000U RNase Inhibitor. The cap analog (3-OMe-GAG) was co-transcribed into the mRNA strands. The IVT reaction was quenched by adding DNase I (100 units; GMP-E127, Novoprotein, China). All mRNAs were purified using the oligo-dT affinity method for subsequent detection (89.7%).

### Preparation and characterization of mRNA-LNPs

mRNA-loaded LNPs were prepared using a microfluidic method. Briefly, the mRNA was dissolved in RNase-free citrate buffer (50 mM, pH 4.0). Lipids including ((4-hydroxybutyl) azanediyl) bis (hexane-6,1-diyl) bis (2-hexyldecanoate), DSPC, cholesterol, and methoxypoly (ethylene glycol)-ditetradecylacetamide were dissolved in anhydrous ethanol at a molar ratio of 46.8:8.9:42.7:1.6. The lipid solution was then mixed with the mRNA solution at a 1:3 ratio using a microfluidic device and further diluted with Tris buffer. Finally, the hydrodynamic size mRNA-LNP was maintained in the range of 116.4 ± 0.65 nm, with PDI of 0.087 ± 0.023 and encapsulation efficiency of 94.5%.

### Antigen expression and western blot analysis

RBD3-Fc mRNA was transfected into HEK293T cells in 6-well plates using Golden Tran-mRNA (Golden Transfer Technology, China; mRNA220521015), according to the manufacturer’s instructions. At 48 h after transfection, the cell lysates and supernatants were collected for western blot analysis. All cell lysates and supernatants were treated with sample buffer and subjected to 12% precast protein gel electrophoresis (Yeasen, China; 36261ES10). Thereafter, the gel was transferred onto PVDF membranes and incubated with 3% no-fat milk for 1 h at room temperature (RT), followed by HRP-linked human Fc antibody (Abcam, ab97225) for 2 h at RT. The samples were then developed using an HRP Substrate (Meilunbio, China, MA0186).

### Optimization of the NFIS parameters for ID delivery

**Figure 2:**
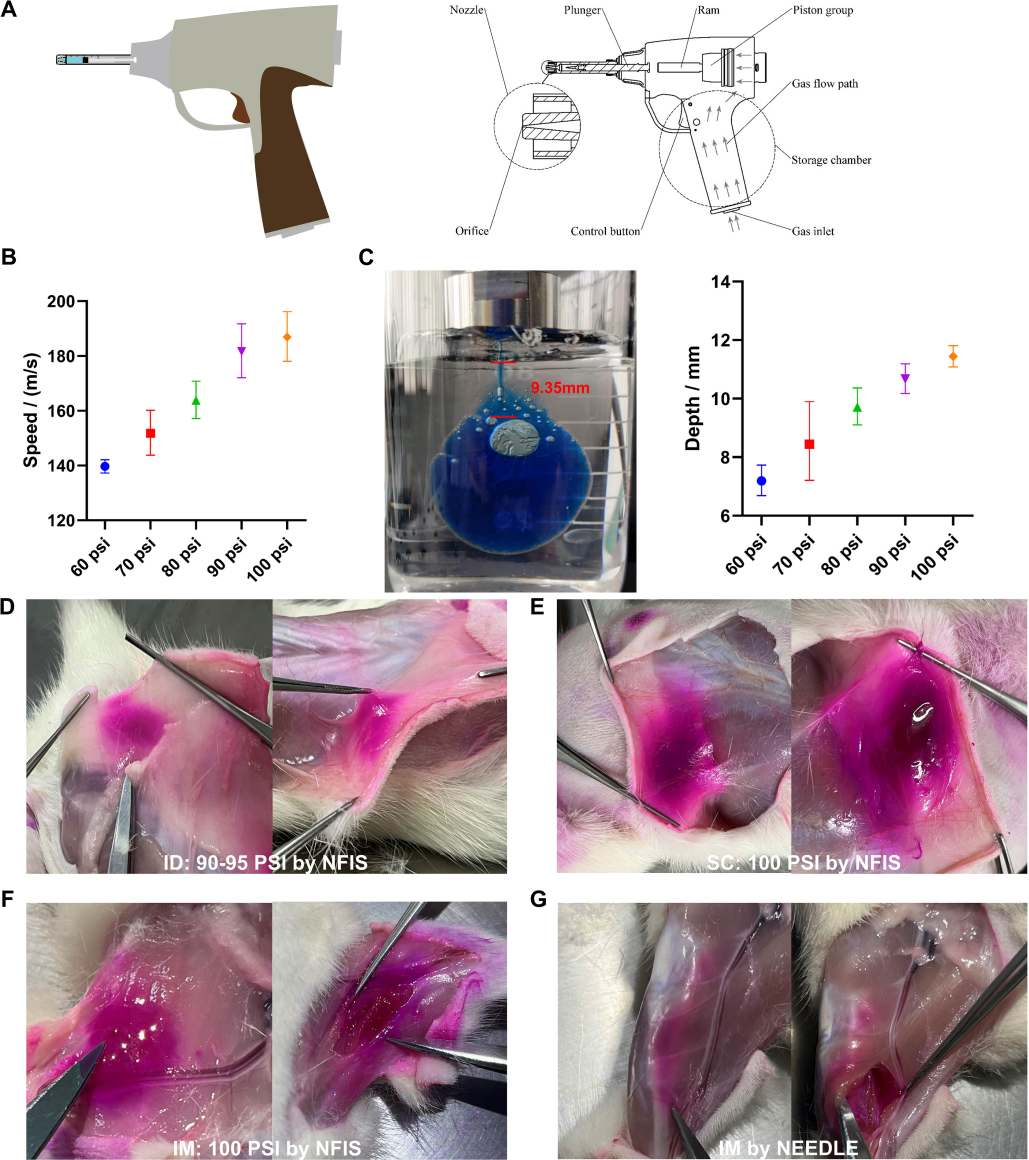
Injection parameters of GV-01 and representative images of mRNA-LNP delivery to rats. **(A)** Gas-propelled NFIS device, GV-01. **(B)** Correlation of pressure and jet velocity. **(C)** Injection depth study using polyacrylamide gel. Representative images of rhodamine-loaded mRNA-LNP delivery via optimum psi for intradermal **(D)**, subcutaneous **(E)**, and intramuscular **(F)** delivery to rats via the NFIS or needle-based IM delivery **(G)**. Administration via the intradermal and subcutaneous route was performed on the back of rats while that via the intramuscular route was performed on the right leg. Data are represented as GMT ± SD **(B, C)**.

The needle-free injector of GV-01, which is a gas-propelled device, is presented in **Figure 2A**. The GV-01 can deliver 0-1 mL of liquid in a single injection. To optimize the device parameters for ID delivery of the vaccine, we first evaluated the driving force-dependent delivery speed and depth of the mRNA-LNP buffer using a polyacrylamide gel. This gel was selected owing to its similar properties to the dermis and subcutaneous tissues in the human skin. Polyacrylamide gels are also transparent and easy to observe. The driving forces (injection pressure) of GV-01 were measured using a high-speed image acquisition system, which was used to observe and track the movement of the push bar. An X190M-64G high-speed camera (Hefei Fuhuang Junda High-tech Information Technology Co., Ltd.) with a resolution of 1280 × 1024 and a small-frame acquisition frame rate of up to 700000 fps was employed in this study. Data were transmitted and collected using network cables with personal computer (PC) terminals. The delivery depth was calculated as the vertical distance between the surface of the gel and the end of the puncture channel at which the solution diffused in all directions, forming a fan-shaped or spherical radiation surface in the gel. Based on the above parameters, injection was carried out on the backs of SD female rats using GV-01 to further optimize the injection pressure and ensure successful ID delivery of the vaccine.

### Safety evaluation

After ID inoculation using GV-01, at the local injection site, a white wheal with a micropore in the center, which was caused by the high-pressure liquid stream, developed on the skin of rats. The sizes of the white wheals were recorded at different time points using a vernier caliper. After inoculation, the animals were examined daily for general signs and local skin reactions at the injection sites. Body weight and food consumption were measured daily and weekly, respectively.

### ELISA

SARS-CoV-2 RBD-specific total immunoglobulin G (IgG) was detected in rat serum using ELISA. The 96-well plates were coated with SARS-CoV-2 prototype RBD (Vazyme, China, CG201) (0.5 μg/well) and incubated at 4°C overnight. The plates were washed three times with 0.05% PBS-T and blocked with 1% BSA at 37°C for 1 h. Diluted serum was added, and the plates were incubated for 2 h at 37°C. After four washes with 0.05% PBS-T, HRP-conjugated goat anti-rat IgG (Absin, China, abs20031) (1:5000) was added to detect antigen-specific total IgG. 3,3′,5,5′-tetramethylbenzidine (TMB) (KPL SeraCare, 5120-0047) was used for the color reaction, and H_2_SO_4_ (2 M) was used to terminate the reaction. The optical density was measured at 450 nm using a microplate reader.

### IgG isotype analysis

To analyze the immunoglobulin isotype profile of sera from the vaccinated rats, standardized ELISA protocols were performed. Briefly, 96-well plates were coated with SARS-CoV-2 prototype RBD (5 mg/mL) and incubated overnight at 4°C. After blocking with 1% BSA at 37°C for 1 h, diluted serum was added and the plates were incubated for 2 h at 37°C. Horseradish peroxidase-conjugated goat anti-rat IgG1 (Sigma, PA1-84708) (1:10000), IgG2a (Sigma, PA1-84709) (1:10000), IgG2b (Sigma, PA1-84710) (1:10000), and IgG2c (Sigma, PA1-84711) (1:10000) were added to detect antigen-specific subclass IgG. The color was developed using TMB and the reaction was terminated using H_2_SO_4_ (2 M). Finally, the absorbance was measured at 450 nm using a microplate reader.

### ELISpot assays

Rat spleens were aseptically extracted 4 weeks after the boost (n = 3). The spleens were washed with RPMI 1640 medium (Gibco, 72400120) and lysed with an ammonium-chloride-potassium (ACK) lysis buffer (Leagene, China; CS0001). Complete medium was then used to stop the lysis reaction. The cell mixture was centrifuged at 300 × g for 5 min to obtain the lymphocytes. Cells were diluted to 2×10^6^ cells/mL and incubated with RPMI 1640 medium (negative control), RBD protein (2.5/5 µg/mL), and PMA (Dakewe, China, positive control) for 48 h. The supernatant was collected and analyzed using the Rat IFN-γ T cell ELISPOT kit (U-CyTech, CT079-PR5) and Rat IL-4 T cell ELISPOT kit (U-CyTech, CT081-PR5), according to the manufacturer’s instructions, to detect IFN-γ and IL-4 secretion. The spots were inspected and counted using an ImmunoSpot S6 Analyzer (Cellular Technology Limited, OH, USA).

### Pseudovirus neutralization assay for SARS-CoV-2

The SARS-CoV-2 pseudoviruses, including D614G, BA.4/5, and B.1.617.2, with Firefly Luciferase (Fluc), were manufactured by Vazyme (China). Neutralizing antibodies prevented pseudovirus-infected cells from expressing Fluc. Briefly, vaccine-immunized serum samples (deactivated at 56°C for 30 min) were serially diluted, incubated with 2×10^4^ TCID50/mL 50 μL/well pseudovirus (1 h at 37°C, in a 5% CO_2_ incubator), and co-cultured with 2 × 10^4^ HEK293-ACE2 cells for 48 h. After reacting with the luminescent substrate, luminescence values were detected. Through a comparison with the luminescence value of the pseudovirus control group, the IC50 (dilution ratio of the antibody when the pseudovirus was suppressed by 50%) was calculated, indicating the neutralizing activity of the antibody against the pseudovirus.

### Single-cell RNA sequencing

CD45+ splenocytes were sorted by flow cytometry. The cells were then loaded into a 10× chromium microfluidic system based on the manufacturer’s guidelines. A set of libraries was obtained from 10× loaded samples: a 3 × gene expression messenger RNA library. The Illumina NovaSeq 6000 sequencing platform and PE150 sequencing mode were used to sequence the completed library; the sequencing amounts should be more than 20 k reads/cell. The scRNA-seq data analysis was performed by NovelBio Bio-Pharm Technology Co., Ltd. using the NovelBrain cloud analysis platform.

### Statistics

Statistical analyses were performed using one-way analysis of variance (ANOVA) or two-way ANOVA in GraphPad Prism 10. Multiple comparisons within a group were performed using Tukey’s multiple comparison test. Differences among groups were considered significant at p < 0.05.

## Results

### Intradermal delivery of mRNA-LNP by GV-01

To record the driving forces (injection pressure) of GV-01 (**Figure 2A**), changes in the movement of the push rod were captured and tracked using a camera during the entire injection process. An analysis software was used to obtain the relationship between the displacement of the push rod and time, as well as the movement speed and jet velocity of the push rod (**Figure 2B**). As shown in **Figures 2B–C**, the injection pressure was positively correlated with the jet velocity and injection depth. With increasing driving force, mRNA-LNP buffer could be delivered to deeper positions. To further confirm injection parameters for ID delivery, SD female rats (age, 9-10 weeks old) were injected with an mRNA-LNP vaccine containing rhodamine B. As shown in **Figures 2D–G**, 90-95 psi was sufficient to deliver the above solution into the ID layer on the backs of rats, while 100 psi enabled SC administration. GV-01 also achieved IM injection into rat legs when the psi was set to 60. Compared with IM administration using needle/syringe, the liquid delivered by GV-01 was more dispersed, aligning with the findings of previous studies (5, 36).

### Design and *in vitro* expression of RBD3-Fc mRNA

An mRNA vaccine candidate (RBD3-Fc) against the SARS-CoV-2 variants was developed based on our previously established mRNA-LNP platform. Spike RBDs from the prototype, BA.4/5 and B1.617.2, were selected as the encoded antigens. In addition, a human IgG1 Fc fragment with a YTE mutation was added to the ORF of the mRNA following the RBD sequence to achieve a long half-life of the antigen and increase its immunogenicity (37–40) (**Figure 1A**). Western blotting was performed using an HRP-conjugated human Fc antibody to evaluate the *in vitro* expression profile of RBD3-Fc mRNA in HEK293T cells. As expected, cell lysates and supernatants from mRNA-transfected HEK293T cells expressed the proteins of interest. Notably, after glycosylation (41), the size of the resultant protein was larger than the expected molecular weight (~77 kD) (**Figure 1B**; **Supplementary Figure 1**). In addition, the antigen was secreted outside the cells, which may aid in the induction of humoral responses through antigen uptake and presentation by APCs at local injection site.

### Safety of RBD3-Fc mRNA vaccines delivered via the ID route using GV-01

**Figure 3:**
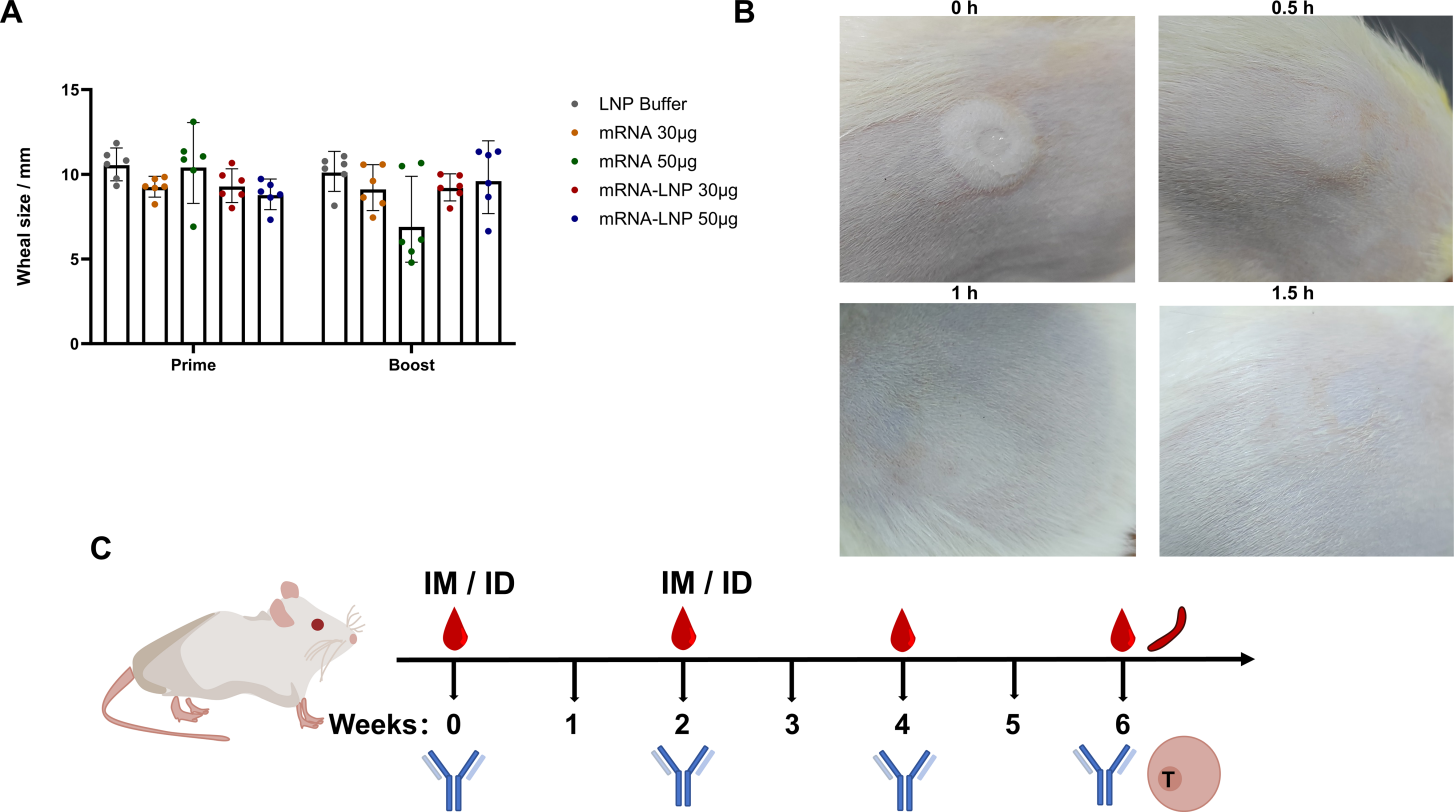
Safety assessment of RBD3-Fc vaccines delivered using the NFIS and via the IM route. **(A)** Wheal sizes at the injection site in each group of rats (n = 6) inoculated with mRNA or mRNA-LNP vaccines using NFIS. **(B)** Representative images of changes in the wheal size after immunization at 1.5 h. Data are representative of two independent experiments. **(C)** Scheme of the immune regimen administered to rats. Rats (six per group) were inoculated with mRNA or mRNA-LNP vaccines via different routes in weeks 0 and 2. IM injection was performed using a needle (29 gauge). Blood samples were collected at weeks 2, 4, and 6 after prime injection for antibody detection. Rats (three per group) were sacrificed at week 6 after prime to obtain spleens for ELISpot and RNA-Seq assays.

To evaluate the safety of the RBD3-Fc mRNA vaccine inoculated using GV-01, side effects resulting from GV-01 injection was evaluated. As expected, wheals with size ranging from 6-12 mm were observed in all groups after inoculation using GV-01 (**Figures 3A, B**). Generally, 8-12 mm wheals indicate successful ID injection. At 1.5 h after immunization, the wheals disappeared in all groups. As for other local side effects, GV-01 resulted in erythema and scab formation, which were more serious than those induced by IM inoculation in the antigen groups (**Supplementary Figures 2A, B**). Notably, the negative control group did not exhibit a similar reaction after ID inoculation, implying that the local reaction was caused by mRNA-LNPs, but not buffer. However, these adverse reactions is transient and disappeared 6 days after inoculation in the groups treated using GV-01. Besides, for either ID or IM inoculation, body weight and food consumption did not significantly differ from those of the negative control, except 50 μg mRNA-LNP via IM, in which transient weight loss occurred after each inoculation, with a significant difference observed (p < 0.05) (**Supplementary Figures 2C–F**). No remarkable systemic side effects were found in the groups vaccinated using GV-01. Overall, ID delivery of the mRNA vaccine using GV-01 was associated with acceptable safety profiles.

### Humoral immune responses of RBD3-Fc mRNA vaccines via the ID route

**Figure 4:**
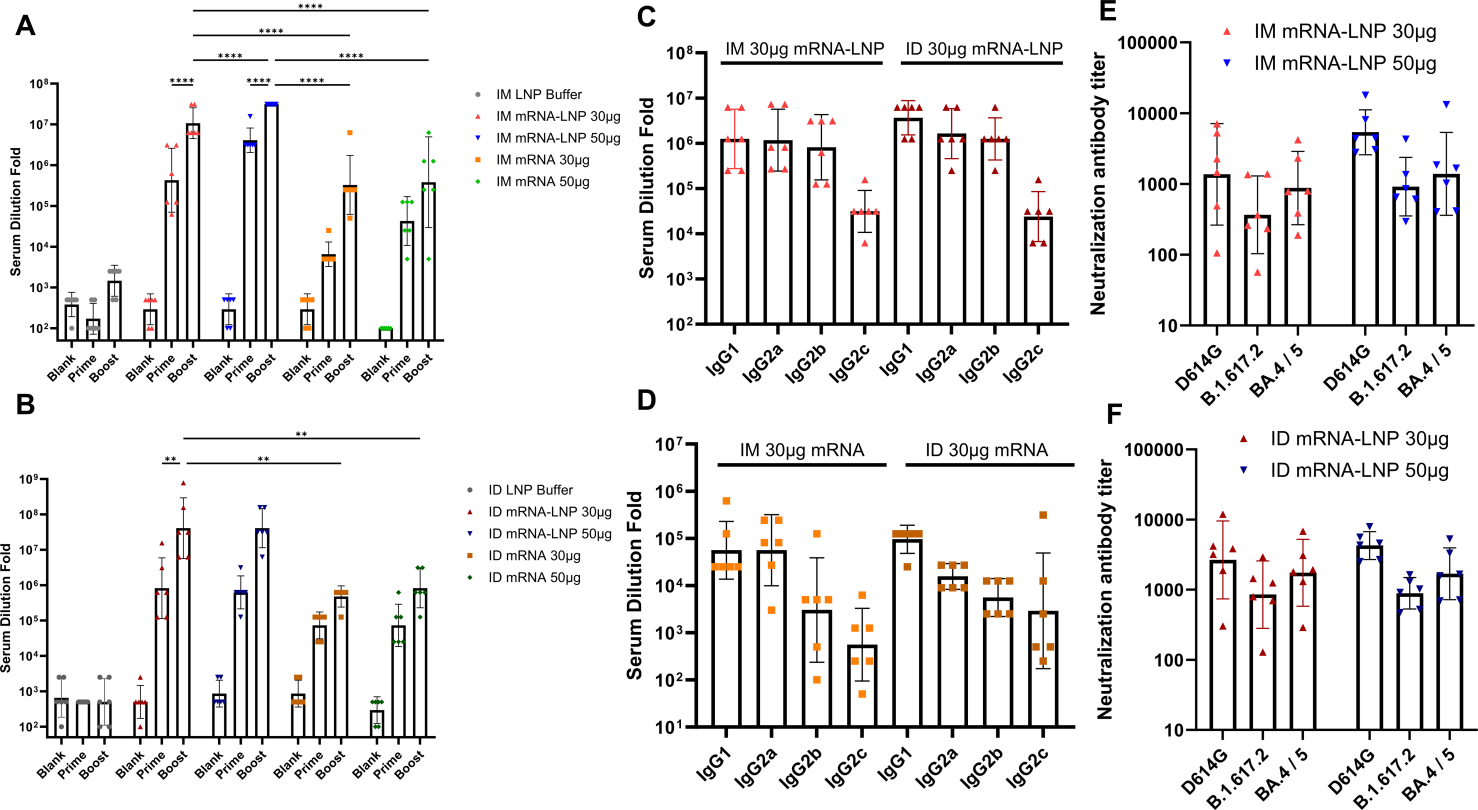
Prototype SARS-Cov-2 RBD-specific IgG antibody **(A, B)** and IgG isotypes **(C, D)** in the sera of rats inoculated with mRNA and mRNA-LNP vaccines via the IM and ID routes after prime and boost injections. Titers of neutralizing antibodies against D614G, Delta, and BA.4/5 strain in sera harvested at week 4 from rats vaccinated with two doses of mRNA-LNP **(E, F)**. All data were presented as geometric means with 95% CI. Significances were assessed by two-way ANOVA with Tukey correction. **P ≤ 0.01, ****P ≤ 0.0001. Data are representative of two independent experiments.

To assess the immunogenicity of the RBD3-Fc mRNA vaccines, rats were vaccinated as shown in **Figure 3C** and **Table 1**. Prototype RBD-specific binding antibodies, IgG isotypes and neutralization antibodies (nAbs) were determined. As shown in **Figures 4A, B**, antigen-specific antibody levels after boosting in all groups were obviously higher than those after priming, suggesting the superior performance of the prime-boost strategy over single injection. In addition, as expected, at the same antigen dose, the groups inoculated with the mRNA-LNP vaccines via two routes displayed significantly higher antibody levels than those inoculated with mRNA vaccines. Higher antibody levels may result from encapsulation with LNP, which protects mRNA from ribozyme degradation during the delivery process *in vivo*. In addition, LNP-mediated endosome escape of mRNA may be more important in improved antigen expression and subsequent antigen presentation. Interestingly, in the IM groups inoculated with either mRNA-LNP or mRNA vaccines, antigen-specific antibody levels increased in an antigen dose-dependent manner, regardless of the prime or prime-boost injections (**Figure 4A**). However, no similar phenomenon was observed in ID groups inoculated with mRNA-LNP vaccines using GV-01, which suggested that ID delivery of 30 µg mRNA-LNP per dose may lead to saturation to some extents. This reasonable speculation was supported by the remarkable antigen dose-dependent increase in the antibody levels in groups inoculated with 5, 10, and 30 µg mRNA-LNP using GV-01 (**Supplementary Figure 3A**). The lower range of antigen doses, corresponding to an mRNA dose-dependent increase in antibody levels of the ID groups, indicated that GV-01-mediated ID delivery of the mRNA-LNP vaccine was superior to needle-mediated IM inoculation. In addition, for the mRNA vaccines, ID delivery generally induced slightly higher RBD-specific antibody levels at the same antigen dose, despite the lack of a significant difference. After 4 weeks of boost, relatively high binding antibodies (**Supplementary Figures 3B, C**) similar with the results of 2 weeks were observed, which suggested antibodies evoked by RBD3-Fc vaccines might be long-lasting.

To identify bias in the immune response induced by mRNA vaccines, we used rat serum to perform IgG isotype assays via ELISA. As shown in **Figures 4C, D**, all mRNA-LNP groups had high levels of antigen-specific IgG1, IgG2a, and IgG2b (titer >10^5^), except for IgG2c (titer >10^2^), than mRNA groups after the boost regardless of inoculation routes. IgG2b and IgG2c are reported to be Th1-related isotypes, while IgG1 and IgG2a are Th2-related isotypes in rats (15, 42, 43). Thus, the above results suggested that mRNA-LNP vaccines elicited both Th1 and Th2 immune responses. In terms of mRNA vaccines, all groups had higher levels of IgG1 than IgG2a, but low IgG2b and IgG2c titers, regardless of the inoculation route and injection technique, indicating an obvious Th2-biased immune response. These results suggested that delivery carrier, but not inoculation route or injection technique, had a more remarkable influence on immune response bias.

Neutralizing antibodies are crucial for preventing COVID-19 infection. Pseudovirus neutralization assays were performed to evaluate the neutralizing antibody levels induced by mRNA vaccines in rats inoculated via the IM or ID route. As shown in **Figures 4E, F**, regardless of the administration route and antigen dose, all groups inoculated with the mRNA-LNP vaccines produced nAbs against the homologous viruses D614G, B.1.617.2, and BA.4/5. In addition, for all groups, the neutralizing antibody levels of BA.4 and D614G were slightly higher than those of B.1.617.2. Interestingly, ID delivery of 30 µg mRNA-LNP led to higher neutralizing antibody levels against all three strains than that induced by IM delivery. However, no statistically significant difference was found between the two routes. When mRNA dose increased to 50 µg, IM delivery of mRNA-LNP showed improved nAbs titers against all three strains, while no obvious increase was found in group inoculated with mRNA-LNP via ID route. As a result, both routes showed comparable capacity in inducing the production of nAbs at the dose of 50 µg mRNA. Besides, as expected, naked mRNA vaccine was less effective at inducing the production of neutralizing antibodies against the above strains than mRNA-LNPs, regardless of the administration route (**Supplementary Figure 3D**). Similar phenomena were observed 4 weeks after the booster (**Supplementary Figure 3E**). Overall, ID inoculation had comparable or better performance in eliciting neutralizing antibody responses against homologous viruses after prime-boost relative to IM inoculation.

### Stronger T cell immune responses of RBD3-Fc mRNA vaccines after inoculation using GV-01

To further assess the T cell immune responses elicited by RBD3-Fc mRNA vaccines delivered via the ID and IM routes, ELISpot assays were performed to detect the secretion of IFN-γ and interleukin-4 (IL-4) in splenocytes (**Supplementary Figures 4A, B**). The delivery of 30 μg of the mRNA-LNP vaccine was more effective at inducing IFN-γ than 30 μg of the mRNA vaccine in two routes, which suggested antigen was able to evoke T cell immune responses against infections. In addition, 30 μg of the mRNA-LNP vaccine via the IM route led to a higher IFN-γ level than delivery via the ID route. In contrast, the secretion levels of IL-4 were very low in all groups, and only one of three rats administered 30 μg of mRNA-LNP via ID route had detectable IL-4 level. These results suggested that the delivery of the mRNA-LNP vaccine via either the IM or ID route elicited T cell immune responses against virus. The above conclusion was also supported by the results of antigen-specific IgG isotype assays.

**Figure 5:**
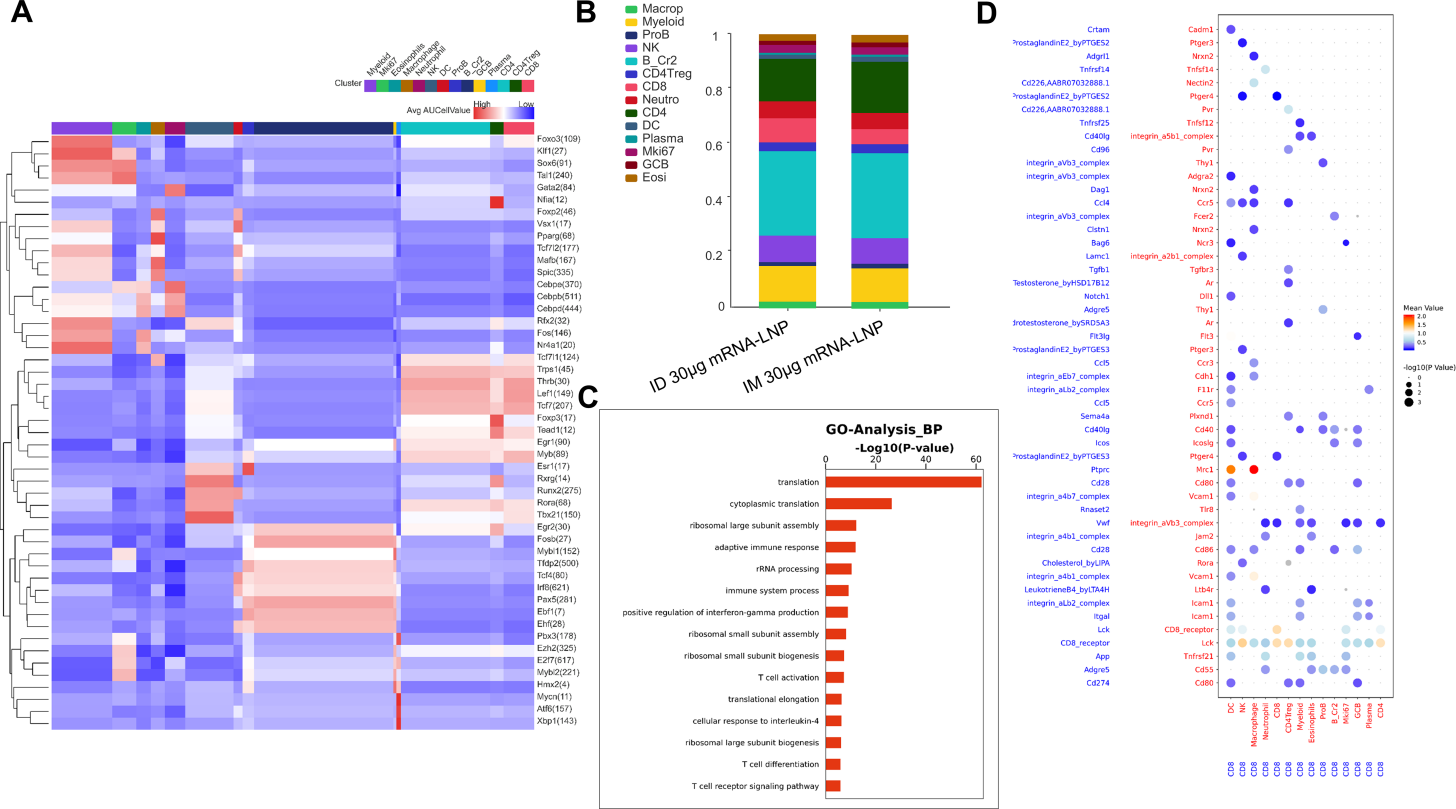
T cell responses evoked after the administration of two doses of 30 μg RBD3-Fc mRNA-LNP vaccines using GV-01. **(A)** Heatmap of the mean value of area under the recovery curve (Aucell) for expression regulation by transcription factors across 14 clusters, as estimated using SCENIC. **(B)** Distinct clusters stacked histogram of the distribution of two groups. GO analysis of biological functions **(C)** and cellphone analysis **(D)** of CD8+ T cells elicited using the NFIS. The dot plot generated using CellPhoneDB highlights the potential ligand-receptor paired association with other immune cells. Dots are colored based on the mean expression of the ligand-receptor pair between two clusters and dot size is proportional to the value of -log10 (P Value). Data are representative of one independent experiment.

To further illustrate the effect of inoculation routes on T cell immune responses induced by the mRNA-LNP vaccine, single-cell RNA sequencing assay (RNA-Seq) was performed using rat splenocytes harvested at week 6. CD45+ splenocytes were separated and captured as immune cells using flow cytometry. Louvain clustering was applied to the filtered and integrated objects and plotted in the uniform manifold approximation and projection (UMAP) space; 14 cell types were identified and manually annotated (**Supplementary Figure 4C**). After merging the two samples, differences between B cells (highly expressing the Cr2 gene) and T cells (CD4 T cells, CD8 T cells) were observed (**Supplementary Figure 4D**). The distribution patterns of these major immune cell subsets in rats were not significantly altered by the vaccination routes; however, both routes led to relatively potent immune responses against COVID-19. To analyze the transcription factors that contribute to immune responses, a single-cell regulatory network inference and clustering (SCENIC) analysis was performed (**Figure 5A**). Tcf7 regulates cell responses to IL-4 and the differentiation of γδT cells, which was in accordance with IL-4 ELISpot results. Upregulation of Tbx21 induces naïve CD4 T cells to acquire Th1 cell-like features (44) to further give the evidence of evoking T cell immunity in line with IFN-γ secretion by ELISpot assays. FoxP3 is a specific marker of regulatory T cells (Tregs) that maintained immune homeostasis in rats (45). Lef1, a 48-kD nuclear protein, is a crucial transcription factor for the proliferation and survival of B and T cells (46), which proved that systematic immune responses after inoculation by NFIS were efficiently aroused against SRSA-Cov-2. Furthermore, the number of CD8 T cells in the group inoculated via the ID route was obviously greater than that in the group inoculated via the IM route (**Figure 5B**); these cells are responsible for adaptive responses and IFN-γ secretion (**Figure 5C**). Moreover, CD8+ T cells displayed robust interactions with DC via the mannose receptor 1 (MRC1) and macrophages via MRC1 and vascular cell adhesion molecule-1 (VCAM-1) (**Figure 5D**), which may suggest vaccination by NFIS increased antigen presentation and improve the ability of CTL and macrophages to clear virus by directly killing infected cells. CD4 T cells had similar biological functions with CD8 T cells to have activity against infections (**Figure 5C**; **Supplementary Figure 4E**). As shown in **Supplementary Figures 4F, G**, antigen-presenting cells like macrophages and DCs first presented processed peptides to CD4 T cells and made them help B cells product antibodies. After GO and KEGG pathway analyses of two groups, different genes were found to be enriched in T cell responses against COVID-19 for T cell differentiation and cytokine secretion, suggesting that systematic immune responses were elicited by both routes, especially using GV-01 (**Supplementary Figures 4H–J**).

To further demonstrate differential T cell immune responses induced by naked mRNA vaccines delivered via different routes, 14 immune cells were annotated and visualized using UMAP software and there were no obvious differences between two groups (**Supplementary Figures 5A, B**). Of note, the number of CD4 T cells induced by 30 μg mRNA delivered using GV-01 was relatively greater than that induced by needles, which was in line with the higher titer of serum antibodies. According to GO analysis (**Supplementary Figure 5C, D**), CD4 and CD8 T cells were responsible for adaptive immune responses. Besides, CD4 T cell also functioned in differentiation and TCR signaling pathway to response to antigens. Overall, the naked mRNA vaccine delivered by two routes was less effective than the mRNA-LNP vaccine in inducing robust systematic immune responses.

## Discussion

The gas-propelled device, GV-01, consisting of a disposable threaded syringe, piston group, and gas inlet, is portable and can deliver a bulk solution (0-1mL) in a single shot. Compared to a syringe-powered device, GV-01 can be used to easily achieve a different route of delivery by modulating the psi parameter for inoculation (**Figure 2**). Due to the wide dispersion pattern of liquids within tissues, NFIS may achieve more efficient delivery of biologics (47, 48), especially mRNA vaccines (1, 5, 49, 50), in a noninvasive manner owing to increased antigen translation and presentation to provoke robust system immune responses (51, 52). In this study, we confirmed that the RBD3-Fc mRNA vaccine delivered intradermally using GV-01 induced comparable or stronger systematic immune responses than those induced by IM delivery using needles (**Figures 4**, **5**), which gives the evidence of replacing syringes to deliver mRNA vaccines with acceptable safety and side effects.

Intradermal delivery of vaccines with 1/10 or 1/5 of fractional doses can induce protective immune responses equivalent to the standard dose delivered intramuscularly in healthy volunteers (53), which the epidermis is rich in LCs and other APCs to translate and present antigens to induce humoral and cell-mediated immune responses (54–56). In this study, no significant dose-sparing effect was observed (**Figure 4**; **Supplementary Figure 3A, D**). However, rats vaccinated by GV-01 with 10 μg mRNA-LNP showed comparable antigen-specific binding antibody titer to that via IM with 50 μg mRNA-LNP 2 weeks after boost, although no statistically significant difference was found. Similar phenomena were also observed in nAbs against D614G. It suggested that 1/5 of fractional doses of RBD3-Fc mRNA-LNP vaccines delivered via ID route may evoke comparable protection to that of full dose of vaccine via IM route against D614G strain infection. In addition, at the same dose of 30 μg, the prototype RBD-specific antibody and CD8 T cell responses in the ID group were higher than those in the IM group, suggested improved humoral and cellular responses. Moreover, the lower range of antigen doses, corresponding to the dose-dependent increase in immunogenicity in the ID groups (5-10 μg for ID delivery vs 5-50 μg for IM delivery), indicated the superiority of ID over IM delivery. Further investigation is needed to illustrate the antigen dose sparing potential of GV-01-mediated ID inoculation in lower antigen dose range. Besides, different animal species, might also influence the evaluation results of dose-sparing effect due to their differential immune systems. In terms of product development and evaluation of the immunization route, larger animals, such as non-human primates may be a better choice as their immune system and induced immune response are highly similar to those of humans.

Even though with the limitations associated with rat models, we gained 14 clusters of immune cells and found key transcription factors of B, CD4 T, CD8 T cells enhanced as shown in RNA-Seq results (**Figure 5**; **Supplementary Figure 4**). The results suggested that mRNA was translated in the cytoplasm and phagocytosed by APCs (e.g. dermal DCs, macrophages), then DCs drained into secondary lymphoid organs where naïve T cells encountered antigens and became effector T cells. CD4 T cells in rats vaccinated with 30 μg mRNA-LNP by GV-01 differentiated into Th1, Th2 and few Th17 subsets. Th1 cells secreted IFN-γ to activate macrophages to eradicate viruses, while Th-2 secreted IL-4 to help B cells produce antibodies in bone marrow, especially nAbs. Interestingly, long-term antibodies were also detected at 4 weeks after boost, which suggested the formation of memory B cells. Of note, a higher number of CD8 T cells was found in the group inoculated with mRNA-LNP ID delivery, suggesting relatively more robust cytotoxic effects to clear viruses. In a rat model, we obtained positive results for the further application of GV-01 in vaccine delivery.

Notably, GV-01 can be used with naked mRNA for ID inoculation to avoid LNP challenges. This delivery had comparable or slightly higher effectiveness than traditional needle-mediated IM inoculation based on the binding titers (> 10^4^), homologous nAbs, and T cell responses (IFN-γ, CD8 T cells). Based on these results, GV-01 may help to deliver at least some of the mRNA directly into the cytoplasm of cells at local injection sites. Skin tissue has relatively more APCs to present translated antigens in MHC-I- and MHC-II-mediated manners than muscle tissue, which is helpful for the induction of immune responses by naked mRNA (**Supplementary Figure 5**). We find different needle-free injectors may have distinct influences on immunogenicity (3, 7, 17, 57, 58). Pyro-drive liquid jet injector (PYRO) (59), which is characterized as bi-phasic jet injection to create a hole and accelerate antigen penetration respectively, can deliver naked mRNA and evoke potent systematic immune responses in rodents and NHPs. In contrast, GV-01 is based on a monophasic jet injection, and therefore may have insufficient antigen penetration and uptake during one gas propulsion process. This may also explain the low effectiveness of naked mRNA relative to that of the mRNA-LNP vaccine (60). mRNA existing outside cells cannot enter cells by themselves without the help of high shear stress due to their inherent large size and highly negative charge. Besides, mRNA inside the cell may be detected by intracellular RNA sensors, including endosomal Toll-like receptors (TLR) and cytoplasmic nucleic acid sensors to drastically degrade (61). Consequently, mRNA that was not delivered into the cytoplasm will be rapidly degraded by extracellular ribozymes (59, 62). Degradation by two ways make mRNA vaccination by GV-01 less efficient to evoke immune responses after prime-boost regimen. Higher mRNA dose and more optimized injection parameters may help to offset the above adverse effect. However, the resultant local and systemic side effects need to be further evaluated.

## Conclusions

In summary, we synthesized RBD3-Fc mRNA and evaluated the immunogenicity and safety of universal RBD3-Fc mRNA vaccines in rats. Both mRNA-LNP vaccine and naked mRNA vaccine showed acceptable safety in rats. Rats vaccinated with mRNA-LNP by GV-01 induced potent systemic immune responses, high titers of homologous nAbs, and strong Th1 and Th2 immune responses. Although GV-01-mediated ID delivery of naked mRNA is less effective than that of mRNA-LNP, ID delivery of the mRNA-LNP vaccine using GV-01 may be an alternative to IM vaccine delivery using traditional needles/syringes. This study indicated that SARS-CoV-2 RBD3-Fc mRNA-LNP may be a promising vaccine against COVID-19, and that GV-01 can be used as an alternative NIFS to deliver both mRNA-LNP and naked mRNA to evoke robust systematic immune responses.

## Data Availability

The data presented in the study are deposited in the GEO repository, accession number GSE288793.
