# Supplementary material for: Intradermal delivery of SARS-CoV-2 RBD3-Fc mRNA vaccines via a needle-free injection system induces robust immune responses in rats
Source: Front Immunol. 2025 Feb 17;16:1530736. doi: 10.3389/fimmu.2025.1530736 (PMC11872709; doi:10.3389/fimmu.2025.1530736)
Supplement: Supplementary file 1 [file DataSheet1.docx]

# Supplementary material


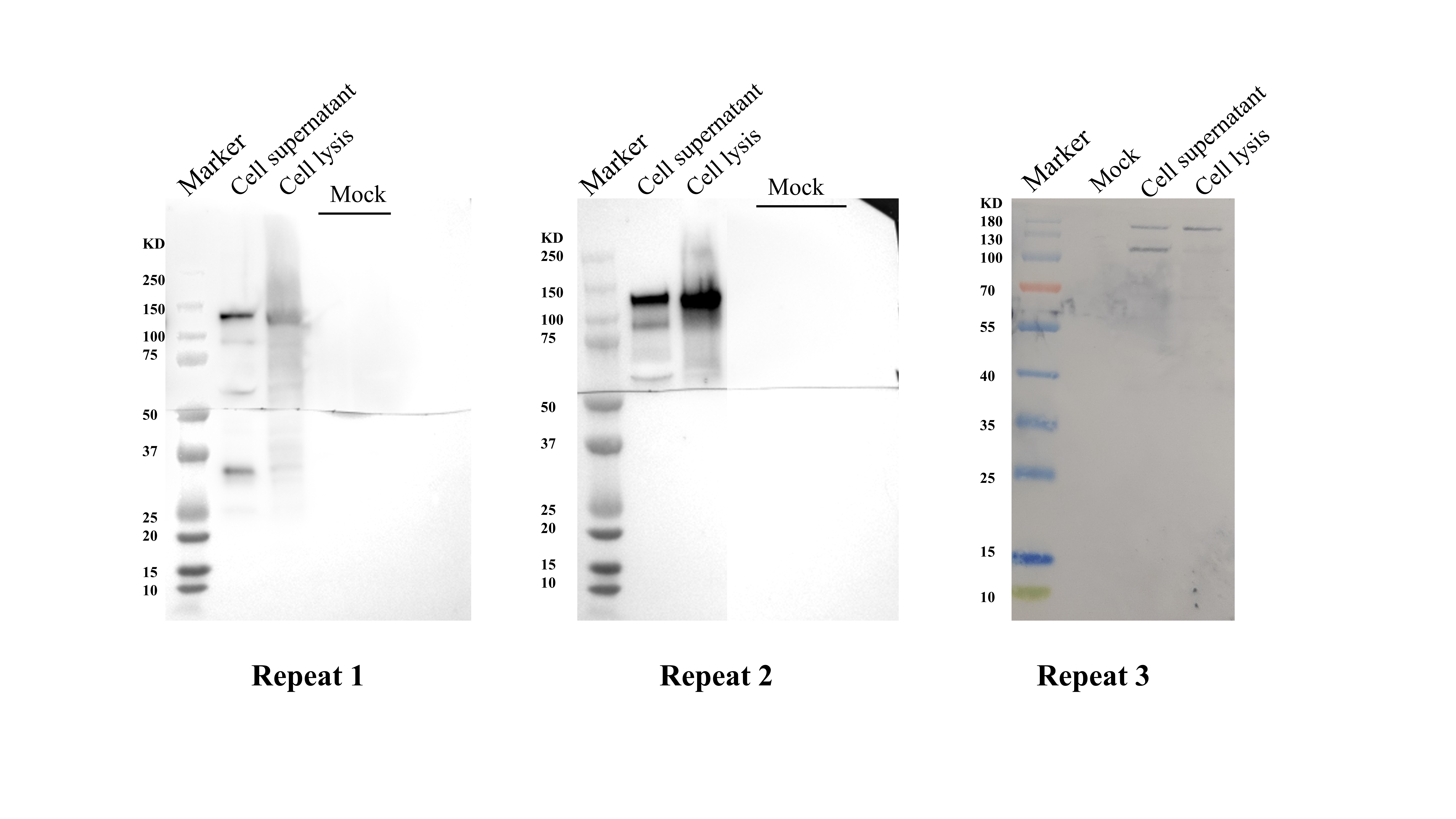


**Supplementary Figure 1. Original western blot for three repeats of RBD3-Fc**. Protein expression of RBD3-Fc mRNA in HEK293T cells detected via western blot.


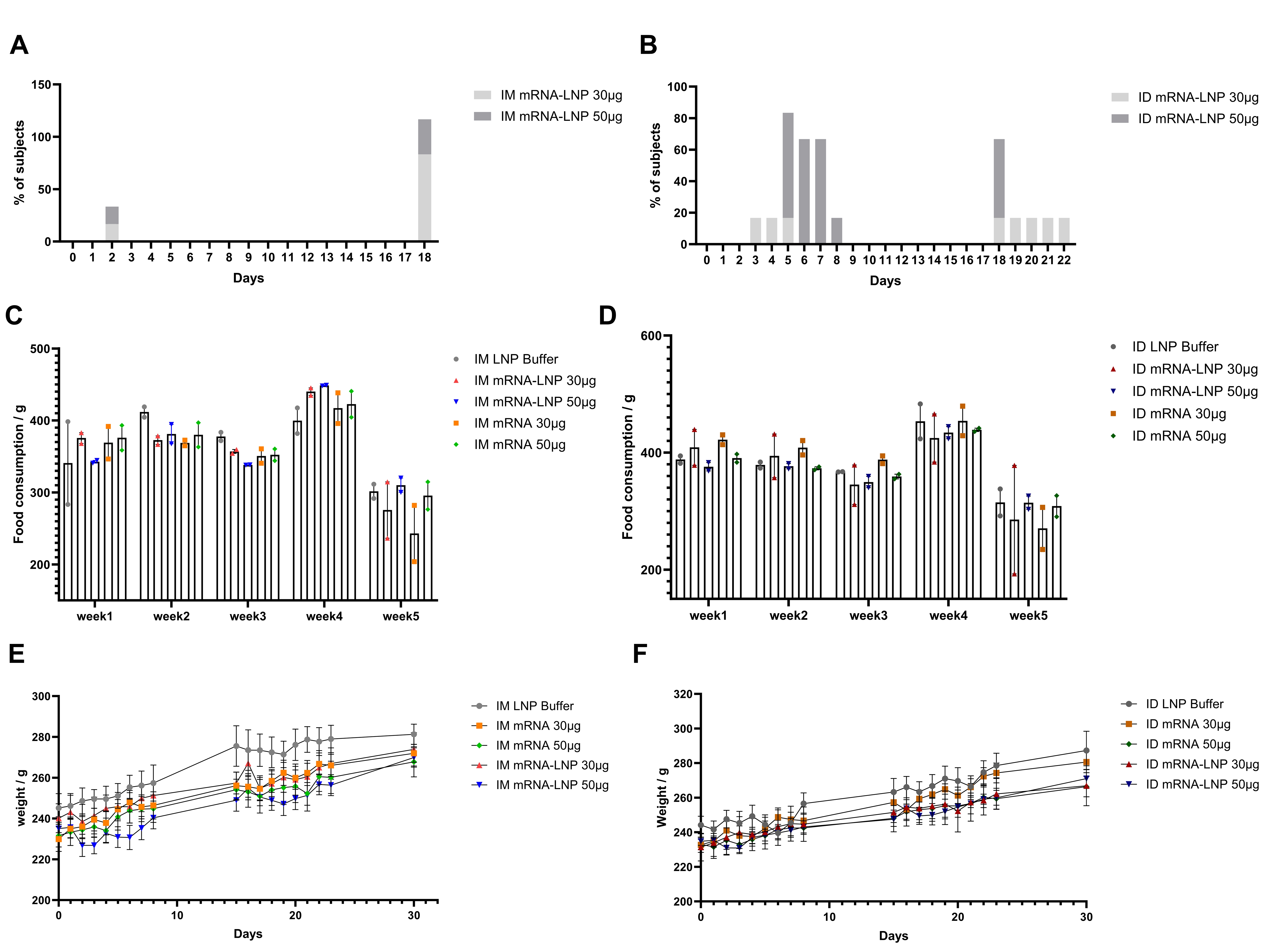


**Supplementary Figure 2. Safety assessment of RBD3-Fc vaccines delivered using the NFIS and via the IM route.** Injection site reaction, including erythema and scab formation, after IM (**A**) and ID (**B**) delivery. Food consumption by rats administered vaccines via the IM (**C**) and ID (**D**) route. Body weight of rats in each group after prime-boost inoculation via the IM **(E)** and ID (**F**) routes until day 30. Data are represented as mean ± SD (A, B) or GMT ± 95% CI (C, D) or mean ± SEM (E, F).


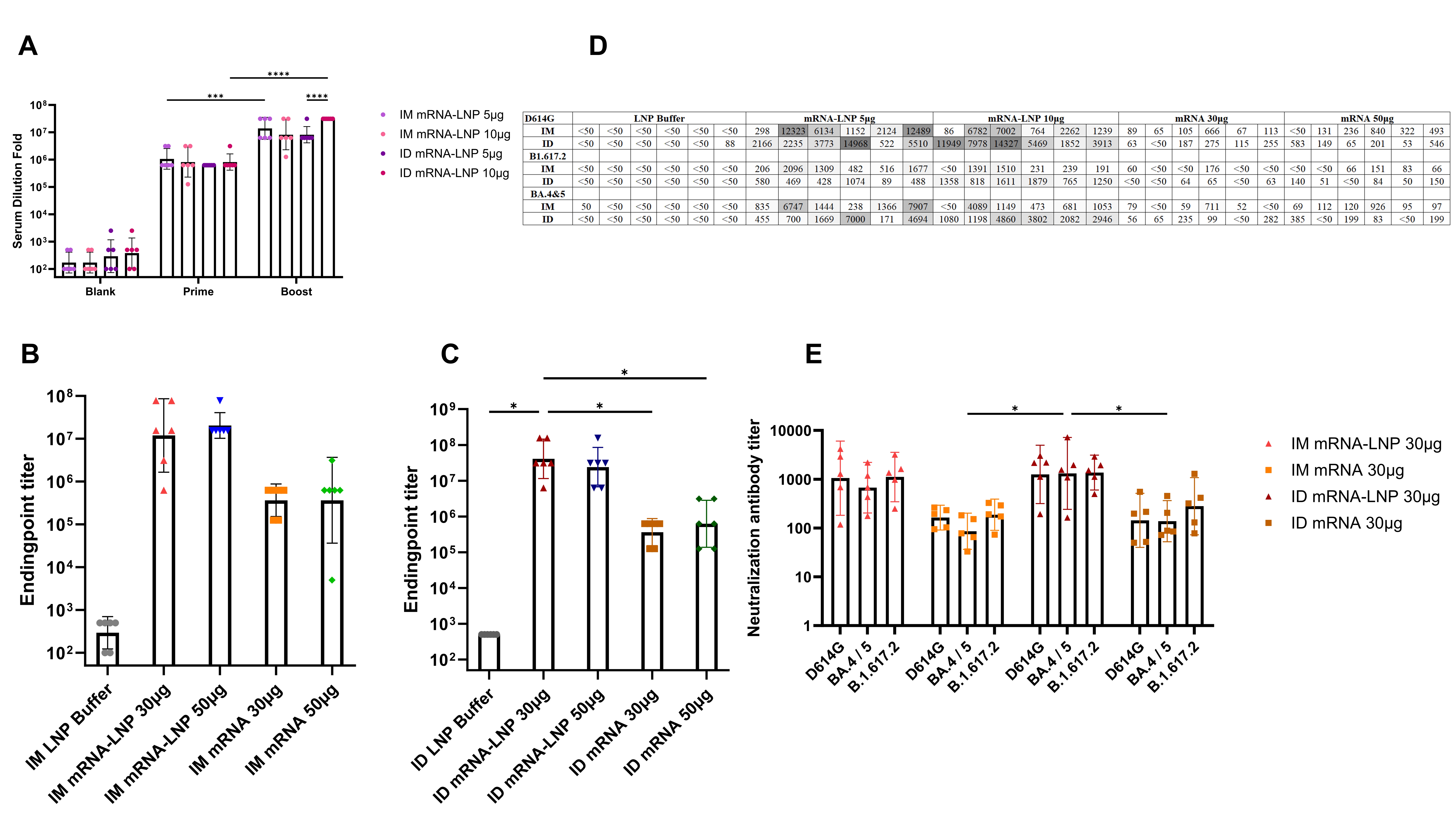


**Supplementary Figure 3. Robust humoral immune responses in rats inoculated with RBD3-Fc vaccines using GV-01.** **(A)** Binding antibodies evoked by 5 μg and 10 μg mRNA-LNP in rats (n = 6) after 2 weeks of boost. Long-term binding antibodies by IM delivery **(B)**, GV-01 **(C).** Serum was collected at 4 weeks after the boost. **(D)** Neutralizing antibodies induced by LNP Buffer, 5 μg, 10 μg mRNA-LNP and 30 μg and 50 μg mRNA detected by the pseudovirus neutralization assay. Serum was collected 2 weeks after the boost. A titer of 50 was set as the limit of detection. **(E)** Neutralizing antibody titers at 4 weeks after the boost. Data are presented as geometric mean with 95% CI and were analyzed using one-way (C, D) or two-way ANOVA (A, E) with Tukey correction. *p ≤ 0.5, **p ≤ 0.01, ***p ≤ 0.001, ****p ≤ 0.0001.





**Supplementary Figure 4. Higher T cell immunity of the RBD3-Fc mRNA-LNP vaccines using GV-01.** Rats were administered two doses of 30 or 50 µg RBD3-Fc mRNA or mRNA-LNP vaccines. Serum and splenocytes were collected at week 6. T cell responses in splenocytes from vaccinated rats (n=3) with prototype RBD proteins (2.5 μg/well) were identified and analyzed using IFN-γ **(A)** and IL-4 **(B)** ELISpot assays. RNA-Seq analysis of UMAP visualization **(C, D)** of CD45+ splenocytes from rats vaccinated with 30 μg mRNA-LNP (n=3) via the NFIS and IM delivery. **(E)** CD4 T cell GO analysis for the NFIS groups and CellPhoneDB analysis with immune cells **(F, G)** as ligands and receptors. **(H)** Biological functions using GO analysis of different genes in the two groups. Top20 gene enrichment in the NFIS group vs IM group of CD4 T cells **(I)** and CD8 T cells **(J)** using KEGG analysis. Data are presented as mean ± SEM.





**Supplementary Figure 5. T cell responses following inoculation with RBD3-Fc naked mRNA vaccine via the NIFS and IM delivery.** Splenocytes were collected 4 weeks after the boost (n=3). Before single-cell sequencing analysis, splenocytes were selected using CD45. **(A)** 14 clusters annotated by UMAP visualization of the two groups immunized via the NFIS and IM delivery. **(B)** Distinct clusters stacked histogram of the distribution of two groups. Different gene enrichments for GO analysis and biological functions of CD8 T **(C)** and CD4 T cells **(D)**.
